# The LINC02041/SRSF1 Axis Facilitates Aerobic Glycolysis and Stemness Maintenance in Hepatocellular Carcinoma

**DOI:** 10.3390/cells15151350

**Published:** 2026-07-27

**Authors:** Mingjiao Cheng, Anqi Cheng, Chenglong Li, Zhibiao Zhang, Tingjiang He, Ludan Zhang, Qianwei Zhao, Jingjing Liu, Weiwei Lin, Jintao Zhang, Fang Xu

**Affiliations:** 1 Henan Institute of Medical and Pharmaceutical Sciences, Zhengzhou University, Zhengzhou 450052, China; cheng13523395518@163.com (M.C.); 18211792895@163.com (A.C.); chenglong199700@163.com (C.L.); htj@gs.zzu.edu.cn (T.H.); 13137790788@163.com (L.Z.); qianwzhao@zzu.edu.cn (Q.Z.); jingliu@zzu.edu.cn (J.L.); linweiwei@zzu.edu.cn (W.L.); 2 Academy of Medical Engineering and Translational Medicine, Tianjin University, Tianjin 300072, China; zhangzhibiao98@tju.edu.cn; 3 Henan Key Medical Laboratory of Tumor Molecular Biomarkers, Zhengzhou University, Zhengzhou 450052, China; 4 Henan Key Laboratory of Tumor Epidemiology, Zhengzhou University, Zhengzhou 450052, China; 5 National Key Laboratory of Metabolism Disorder and Esophageal Cancer Prevention & Treatment, Zhengzhou University, Zhengzhou 450052, China

**Keywords:** HCC, glycolysis, stemness, LINC02041, STAT3, SRSF1

## Abstract

Hepatocellular carcinoma (HCC) remains one of the most aggressive and lethal malignancies worldwide, with high rates of metastasis and recurrence contributing to its poor prognosis. There is an urgent need to elucidate the molecular mechanisms driving HCC progression and to develop effective therapeutic strategies. Metabolic reprogramming, especially aerobic glycolysis known as the Warburg effect, is a well-established hallmark of cancer. Concurrently, cancer stem cells (CSCs) play crucial roles in tumor initiation, therapy resistance, and recurrence. However, the involvement of long non-coding RNAs (lncRNAs) in linking metabolic alterations and stemness remains poorly understood. In this investigation, we identified LINC02041 as a significantly upregulated lncRNA in HCC tissues and demonstrated its oncogenic role in promoting cell proliferation. We found that STAT3 transcriptionally activates LINC02041 expression. Mechanistically, LINC02041 enhances the stability of SRSF1 protein by suppressing its ubiquitin-mediated degradation, thereby facilitating HCC cell proliferation, migration, glycolytic metabolism, and acquisition of stem-like properties. Our findings delineate a novel STAT3/LINC02041/SRSF1 regulatory axis that coordinately modulates glycolytic reprogramming and stemness maintenance in hepatocarcinogenesis. This study not only advances our understanding of HCC pathophysiology but also identifies LINC02041 as a promising prognostic biomarker and a compelling therapeutic target for novel therapeutic strategies against this aggressive malignancy.

## 1. Introduction

Hepatocellular carcinoma (HCC) remains one of the most aggressive malignancies worldwide and represents a major cause of cancer-related mortality. Despite advances in diagnostic approaches and therapeutic strategies, the patient prognosis with advanced HCC remains poor due to frequent recurrence, metastasis, and acquired therapeutic resistance [1,2]. Therefore, identifying novel molecular regulators involved in HCC progression is essential for improving diagnostic strategies, prognostic assessments, and individualized treatments.

Currently, systemic therapies for unresectable advanced HCC primarily encompass molecular targeted therapies and immune-based approaches. The multikinase inhibitors sorafenib and lenvatinib are established first-line systemic treatments, whereas regorafenib and cabozantinib are approved as subsequent therapeutic options after sorafenib failure. In addition, immune checkpoint inhibitor-based therapies, including atezolizumab plus bevacizumab and durvalumab plus tremelimumab, have expanded the therapeutic landscape for patients with advanced HCC [2,3,4]. However, acquired resistance and tumor recurrence remain major obstacles limiting the long-term efficacy of these therapies. Increasing evidence indicates that cancer stem cells (CSCs), a subpopulation of tumor cells characterized by self-renewal capacity and differentiation potential, play a pivotal role in tumor recurrence, metastasis, and therapeutic resistance [5,6]. In HCC, CSC populations have been implicated in resistance to molecular targeted therapies through enhanced survival signaling, metabolic adaptation, and maintenance of stemness-associated properties [7,8]. Therefore, understanding the molecular mechanisms that regulate CSC characteristics and therapy resistance is critical for developing more effective therapeutic strategies.

Metabolic reprogramming is a fundamental feature of cancer progression, enabling tumor cells to adapt to increased energy demands and unfavorable microenvironments [9]. Aerobic glycolysis, known as the Warburg effect, is characterized by increased glucose uptake and lactate production despite sufficient oxygen availability, representing a major metabolic alteration that supports tumor growth and metastatic progression [10,11,12,13]. Beyond providing energy and biosynthetic intermediates, metabolic remodeling also contributes to tumor cell plasticity and the maintenance of CSC properties [14]. Moreover, enhanced glycolytic activity has been reported to contribute to therapeutic resistance, including resistance to molecular targeted therapies in HCC [15]. However, the molecular mechanisms linking cancer-associated regulators to glycolytic reprogramming and CSC maintenance in HCC remain incompletely understood.

Accumulating evidence demonstrates that long non-coding RNAs (lncRNAs) participate extensively in cancer development through diverse molecular mechanisms [16,17]. LncRNAs are transcripts typically longer than 200 nucleotides that lack protein-coding potential and regulate various biological processes via mechanisms such as transcriptional regulation, chromatin remodeling, modulation of RNA stability, and protein interactions [18,19,20,21,22]. In contrast to protein-coding genes, lncRNAs exhibit highly context-dependent expression patterns and play critical roles in tumor proliferation, apoptosis, invasion, metastasis, metabolism, and stem cell maintenance [18,19]. Although numerous cancer-associated lncRNAs have been identified, the biological functions and molecular mechanisms of many lncRNAs remain poorly understood.

In our previous study, transcriptome analysis of HCC tissues and corresponding adjacent non-tumor tissues identified multiple dysregulated lncRNAs, including LINC02041 [23]. Previous studies have demonstrated that LINC02041 (also annotated as RP11-567G11.1) promotes the migration and invasion of tumor cells in pancreatic cancer and renal cell carcinoma [24,25]. However, its biological function and underlying regulatory mechanisms in HCC remain to be elucidated.

SRSF1 (serine/arginine-rich splicing factor 1), a prototypical member of the SR protein family, is an oncogenic splicing factor involved in tumor development and progression [26]. Previous studies have shown that SRSF1 promotes tumorigenesis through the regulation of alternative splicing, cell proliferation, apoptosis, and metastatic signaling pathways [26,27]. However, it remains unclear whether lncRNAs regulate SRSF1 protein stability and contribute to HCC progression.

In this study, we investigated the biological functions and underlying molecular mechanisms of LINC02041 in HCC. LINC02041 was upregulated in the TCGA-LIHC cohort and in an independent clinical tissue cohort. Functional assays demonstrated that LINC02041 promotes HCC cell proliferation, migration, invasion, glycolytic activity, and stem-like properties. Mechanistically, our findings support a regulatory model in which STAT3 transcriptionally activates LINC02041, while LINC02041 increases SRSF1 protein stability by reducing its ubiquitin-mediated degradation. These findings identify LINC02041 as a candidate biomarker and therapeutic target that requires further clinical validation in HCC.

## 2. Materials and Methods

### 2.1. Cell Lines and Culture Conditions

SNU449, SMMC7721, HCCLM3, and 293T cell lines were obtained from the Cell Bank of the Chinese Academy of Sciences (Shanghai, China). Huh-7 cells were purchased from Wuhan Sevier Biotechnology Co. The 293T cell line and human HCC cell lines, including HCCLM3, Huh-7, and SMMC7721, were cultured in Dulbecco’s Modified Eagle Medium (DMEM, high glucose; Gibco, Grand Island, NE, USA) supplemented with 10% fetal bovine serum (FBS). SNU449 cells were maintained in RPMI-1640 medium (Gibco, Grand Island, NE, USA) supplemented with 10% FBS. All cells were cultured at 37 °C in a humidified atmosphere containing 5% CO_2_. Cells were routinely screened for mycoplasma contamination using the MycoAlert™ Mycoplasma Detection Kit (Lonza, Basel, Switzerland) according to the manufacturer’s instructions, and all cell lines were confirmed to be mycoplasma-free before experiments. All experiments were performed using cells between passages 5 and 15.

### 2.2. Real-Time Fluorescence Quantitative PCR

Total RNA was extracted using TRIzol reagent (Servicebio, CR2410066, Wuhan, China) according to the manufacturer’s instructions. cDNA was synthesized using the PrimeScript RT Reagent Kit (TaKaRa Clontech, RR036, Dalian, China). Quantitative real-time PCR was performed using ChamQ™ Universal SYBR^®^ qPCR Master Mix (Biosharp, BL697A, Shanghai, China) with a QuantStudio™ 5 Real-Time PCR System (Thermo Fisher Scientific, Waltham, MA, USA). Primer sequences for all target genes are listed in Supplementary Table S1. The thermal cycling conditions were as follows: 95 °C for 2 min; followed by 40 cycles of 95 °C for 15 s, 60 °C for 30 s, and 72 °C for 30 s. Relative gene expression levels were calculated using the 2^−ΔΔCt^ method with β-actin as the internal control [28]. All qPCR reactions were performed in technical triplicates, and each experiment was independently repeated at least three times.

### 2.3. Chromatin Immunoprecipitation (ChIP) Assays

Chromatin immunoprecipitation (ChIP) assays were performed using the BeyoChIP™ Enzymatic ChIP Assay Kit (Protein A/G Magnetic Beads) (Beyotime, P2083S, Shanghai, China) according to the manufacturer’s instructions. Briefly, 5 × 10^6^ hepatocellular carcinoma cells were crosslinked with 1% formaldehyde for 10 min at room temperature and subsequently quenched with 125 mM glycine for 5 min. Cells were subsequently lysed, and chromatin was fragmented via micrococcal nuclease (MNase) digestion according to the manufacturer’s protocol.

Before immunoprecipitation, 2% of the fragmented chromatin was reserved as the input control. The remaining chromatin was incubated overnight at 4 °C with 4 μL of an anti-STAT3 antibody (Proteintech, 10253-2-AP, Wuhan, China) or normal rabbit IgG, which served as the nonspecific immunoprecipitation control. The immune complexes were captured using Protein A/G magnetic beads, followed by sequential washing, reverse crosslinking, and DNA purification according to the manufacturer’s instructions.

The SOCS3 promoter was examined as a positive-control locus for STAT3 binding, whereas a distal region located approximately 1.4 kb upstream of the examined LINC02041 promoter region was examined as a negative-control genomic locus. These control loci were used to verify the validity and specificity of the ChIP assay. The purified DNA was analyzed by quantitative PCR. ChIP enrichment was expressed as the percentage of input DNA (% input) and calculated using the input-adjusted threshold cycle values. The primer sequences are listed in Supplementary Table S1.

### 2.4. Metabolic Assays

Glycolytic activity was assessed via quantification of glucose consumption and lactate production. After transfection, cells were seeded in 24-well plates at a density of 2 × 10^5^ cells/well and incubated for 48 h. Subsequently, the conditioned medium was collected for metabolic analysis. Glucose consumption and lactate production were measured using the Cell Glucose Assay Kit (Bestbio, BB-47305-500T, Shanghai, China) and the Lactic Acid Assay Kit (Bestbio, BB-47223-500T, Shanghai, China), respectively. Following the manufacturer’s protocols. For glucose quantification, absorbance was measured at 505 nm against a 5.55 mmol/L glucose standard. For lactate quantification, samples were diluted 10-fold, and absorbance was measured at 530 nm relative to a 3 mmol/L lactate standard. Each experimental group included 3–4 replicate wells, and metabolite concentrations were calculated according to the standard curves provided in the kit instructions.

### 2.5. Cell Migration and Invasion

Cell migration and invasion assays were performed using Transwell inserts with 8-μm pore polycarbonate membranes (Corning, Corning, NY, USA). Cells were serum-starved overnight, harvested, and resuspended in serum-free DMEM. A total of 1.2 × 10^5^ cells suspended in 200 μL serum-free DMEM were seeded into the upper chamber of each insert, while 600 μL of DMEM supplemented with 20% fetal bovine serum (FBS) was added to the lower chamber as a chemoattractant. After incubation for 24 h, non-migrated cells on the upper surface of the membrane were gently removed using a cotton swab. Cells that had migrated to the lower surface of the membrane were fixed with 4% paraformaldehyde for 30 min, stained with 1% crystal violet aqueous solution (Leagene, DZ0053, Beijing, China) for 30 min, and washed with PBS. Images were captured using an inverted microscope at 200× magnification. Five randomly selected non-overlapping fields were analyzed for each insert, and migrated cells were quantified using ImageJ software (version 1.52a, NIH, Bethesda, MD, USA) [29]. Statistical analyses were performed using GraphPad Prism software (version 9.0.0, Boston, MA, USA). Each experimental group included three replicate inserts.

For invasion assays, the upper surface of each insert was coated with 30 μL Matrigel matrix (BD Biosciences, 356234, San Jose, CA, USA) diluted 1:6 in serum-free DMEM and incubated to allow polymerization before cell seeding. All subsequent procedures followed the protocol established for the migration assay.

### 2.6. Sphere Formation Assays

Sphere formation assays were performed using ultra-low attachment 6-well plates (Corning, NY 3471, USA). Cells were seeded at a density of 5000 cells per well in sphere formation medium, which consisted of DMEM supplemented with 50 ng/mL bFGF, 50 ng/mL EGF, and 2% (*v*/*v*) B27 supplement. Cells were maintained at 37 °C under 5% CO_2_ for 10 days, replacing half of the medium volume every 3 days. Spheres were defined as cellular aggregates with a diameter of ≥40 μm. At the endpoint, images were captured from 3–5 randomly selected fields per well using an inverted microscope, and sphere number and diameter were quantified via ImageJ software [29].

### 2.7. Patient Samples

Tumor tissues and paired adjacent non-tumor tissues were collected from 12 patients with hepatocellular carcinoma who underwent surgical resection at the First Affiliated Hospital of Zhengzhou University. The enrolled patients included 8 males and 4 females, with ages ranging from 45 to 72 years. According to the AJCC 8th edition staging system, 5 patients were classified as stage I, 4 patients as stage II, and 3 patients as stage III. None of the patients received preoperative anticancer treatment, including chemotherapy, radiotherapy, targeted therapy, or immunotherapy, before surgery.

Tissue samples were collected immediately after surgical resection, rapidly snap-frozen in liquid nitrogen, and stored at −80 °C until further analysis. The study protocol was approved by the Ethics Committee of Zhengzhou University (No. YLL-019) and was conducted in accordance with the Declaration of Helsinki. Written informed consent was obtained from all patients before sample collection. The approval date is 1 January 2023.

### 2.8. Statistical Analysis

Statistical analysis was conducted using GraphPad Prism 9 software and data were presented as the mean ± SD from three independent experiments. Student’s *t*-test and one-way ANOVA were used to analyze the data. A *p*-value less than 0.05 was considered statistically significant.

### 2.9. RNA Sequencing and Functional Annotation Analysis

Three LINC02041-knockdown Huh7 samples and three negative-control samples underwent paired-end 150 bp RNA sequencing on the BGISEQ platform. Clean reads were aligned to the human reference genome (GRCh38.p13) using HISAT and to reference gene sequences using Bowtie2. Differentially expressed genes were identified using DESeq2, filtered at q < 0.05, and subjected to GO and KEGG functional annotation via the BGI analysis pipeline.

### 2.10. Animal Experiments and Subcutaneous Xenograft Model

Male BALB/c nude mice (4–6 weeks old, 18–22 g) were used for subcutaneous xenograft experiments to evaluate the role of LINC02041 in HCC tumor growth in vivo. Mice were housed under specific pathogen-free (SPF) conditions with controlled temperature (22 ± 2 °C), relative humidity (50–60%), and a 12 h light/dark cycle, with free access to food and water.

Mice were randomly assigned to the indicated experimental groups, with five mice in each group.

For the LINC02041 overexpression experiment, mice were subcutaneously injected with SMMC7721 cells stably overexpressing LINC02041 or corresponding vector control cells. For the LINC02041 knockdown experiment, mice were injected with HCCLM3 cells stably expressing LINC02041 shRNA or corresponding control cells. For the rescue experiment, mice were injected with HCCLM3 cells subjected to LINC02041 knockdown alone or combined with SRSF1 overexpression.

Tumor volume was measured every three days after tumor inoculation using the formula: tumor volume = (length × width^2^)/2. At the experimental endpoint, mice were euthanized, and xenograft tumors were collected, weighed, and processed for subsequent analyses.

All animal experiments were approved by the Institutional Animal Care and Use Committee of Zhengzhou University (Approval No. 2025-YYY-054) and were conducted in accordance with relevant guidelines for laboratory animal care. Blinding was not performed during the animal experiments.

## 3. Results

### 3.1. LINC02041 Is Highly Expressed in Cancer Tissues from HCC Patients and Is Associated with Poorer Patient Overall Survival

**Figure 1:**
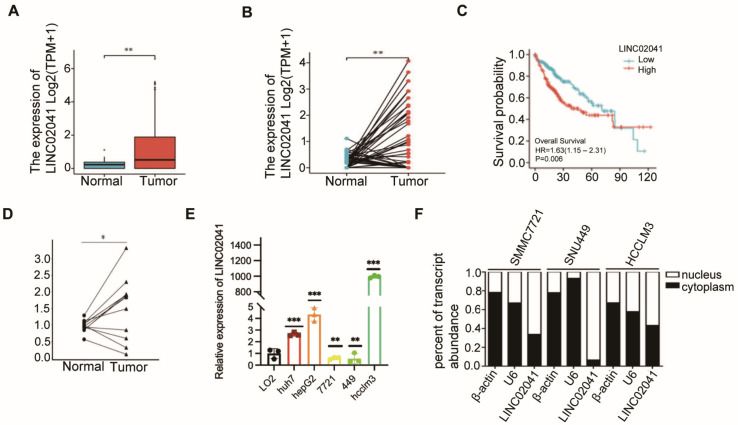
LINC02041 is elevated in human HCC and correlates with an unfavorable prognosis. (**A**,**B**) show expression of LINC02041 in adjacent non-tumor and tumor samples from HCC patients analyzed by the TCGA database. (**C**) Survival curves showing overall survival of HCC patients with different expression levels of LINC02041. (**D**) RT-qPCR analysis of LINC02041 expression in tumor and adjacent non-tumor tissues from 12 paired clinical HCC samples. Dots represent the normal group, and triangles represent the tumor group. (**E**) RT-qPCR analysis of relative RNA expression of LINC02041 in five HCC cell lines. (**F**) RT-qPCR analysis of LINC02041 subcellular localization in three HCC cell lines. * *p* < 0.05; ** *p* < 0.01; *** *p* < 0.001.

Analysis of the TCGA-LIHC dataset [30] revealed LINC02041 expression profiles in both unpaired and paired normal and tumor samples from HCC patients. We also assessed the impact of LINC02041 expression levels on patient prognosis. Compared to adjacent non-cancerous tissues, LINC02041 expression was higher in HCC tissues. Patients with high LINC02041 expression had a poorer prognosis (Figure 1C). The analysis included 50 normal and 374 tumor samples (Figure 1A), as well as 50 paired normal and tumor samples (Figure 1B). We next measured the RNA expression levels of LINC02041 in 12 paired human HCC and adjacent non-tumor tissues using RT-qPCR. The results were consistent with the TCGA data (Figure 1D). The expression of LINC02041 in the normal human hepatocyte cell line LO2 and HCC cell lines was analyzed by RT-qPCR. As shown in Figure 1E, LINC02041 expression was significantly down-regulated in the HCC cell lines SMMC7721 and SNU449 compared to LO2; however, in Huh7 and HCCLM3 cells, LINC02041 expression was significantly up-regulated. RT-qPCR analysis of LINC02041 expression levels in the nucleus and cytoplasm fractions of SNU449, SMMC7721 and Huh7 cells revealed that LINC02041 is predominantly localized to the cytoplasm.

### 3.2. LINC02041 Promotes HCC Cell Proliferation, Migration and Invasion

**Figure 2:**
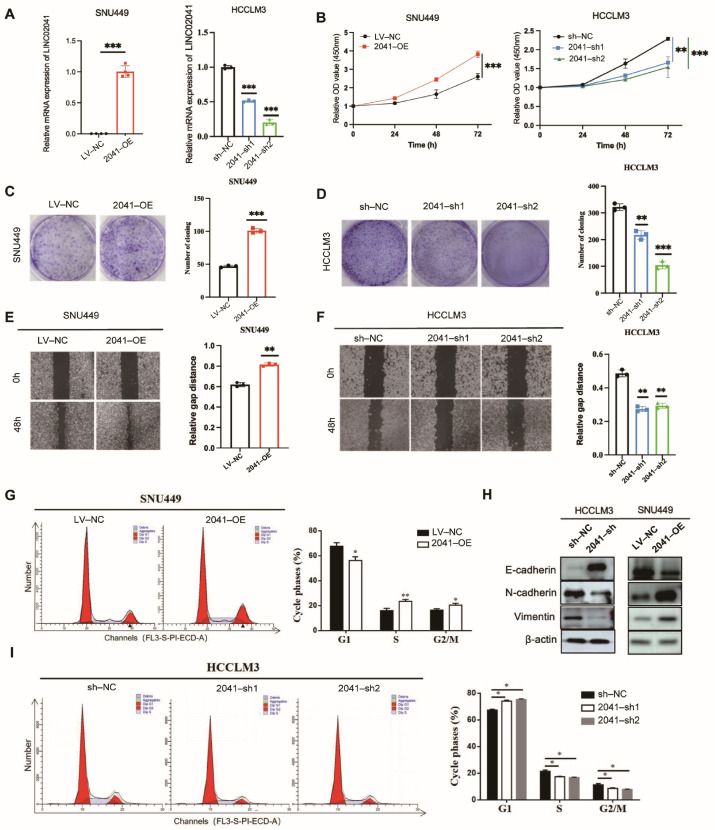
LINC02041 promotes the proliferation, migration, and invasion of HCC cells in vitro. (**A**) RT-qPCR was used to detect RNA expression of LINC02041 in SNU449 and HCCLM3 cells. (**B**) CCK-8 assay was performed to assess the effect of knockdown or overexpression of LINC02041 on the proliferation ability of HCC cells. (**C**,**D**) Colony formation assays were conducted to evaluate the effect of knockdown or overexpression of LINC02041 on the colony formation ability of HCC cells. Cell proliferation assay of SNU449 and HCCLM3 cells under different treatments. In the SNU449 cell line, black dots represent the LV–NC group, and red squares represent the 2041–OE group. In the HCCLM3 cell line, black dots represent the sh–NC group, blue squares represent the 2041–sh1 group, and green triangles represent the 2041–sh2 group. (**E**,**F**) Wound healing assay was used to assess the cell migration of HCCLM3 and SNU449 cells with knockdown or overexpression of LINC02041 and their control cells. (**G**,**I**) Flow cytometry assay was performed to analyze the differences in the distribution of HCC cells at each cell cycle phase between SNU449 and HCCLM3 cells with overexpressing or knock down of LINC02041 and their corresponding control cells. (**H**) Western blot analysis of E-cadherin, N-cadherin and Vimentin protein expression in HCCLM3 cells with knockdown or overexpression of LINC02041 and in SNU449 cells with their control cells. * *p* < 0.05; ** *p* < 0.01; *** *p* < 0.001.

To investigate the effect of LINC02041 on HCC development, SMMC7721 and SNU449 cell lines with low expression of LINC02041 were selected and transfected with LINC02041-overexpressing viruses or their corresponding empty vector negative controls. Meanwhile, HCCLM3 cells with high expression of LINC02041 were transfected with LINC02041 knockdown viruses or their respective empty vector controls (Figure 2A). The effect of LINC02041 knockdown or overexpression on the proliferative ability of HCC cells was assessed by CCK-8 assay. As shown in Figure 2B, the proliferation capacity of cells in the LINC02041 overexpression group was significantly enhanced over time compared with the control group. Subsequently, colony formation assays were performed, demonstrating that overexpression of LINC02041 significantly promoted the colony-forming ability of HCC cells, while knockdown of LINC02041 significantly inhibited this ability (Figure 2C,D).

The effect of LINC02041 on the migration ability of HCC cells was evaluated by wound healing assays. The results shown in Figure 2E,F indicated that LINC02041 significantly promoted the migration ability of HCC cells. Cell cycle analysis by flow cytometry revealed that knockdown of LINC02041 induced cell cycle arrest at the G1 phase in HCC cells (Figure 2G,I). Western blot analysis (Figure 2H) showed that overexpression of LINC02041 downregulated E-cadherin while upregulating N-cadherin and Vimentin compared to control cells, suggesting an enhanced metastatic potential of HCC cells.

### 3.3. LINC02041 Promotes Tumor Growth In Vivo

**Figure 3:**
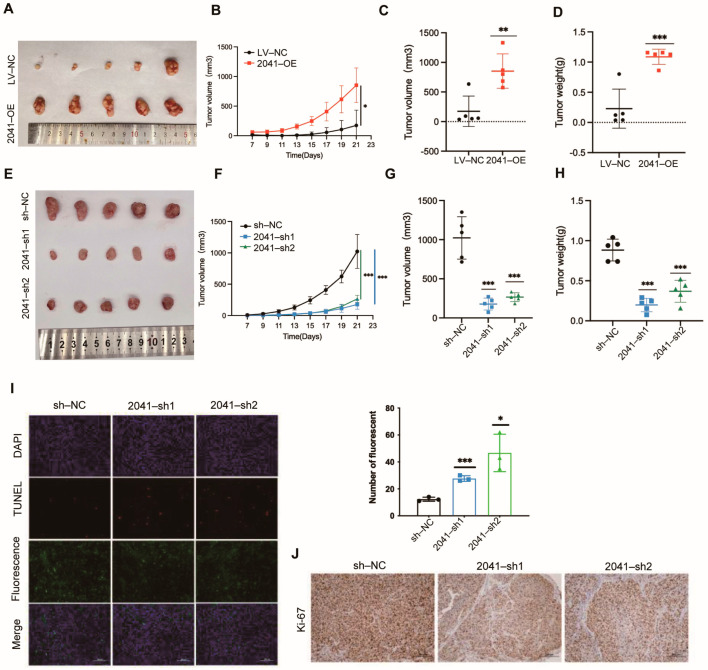
LINC02041 promotes tumor growth of HCC in mice in vivo. (**A**) Nude mice were dissected and photographed 21 days after subcutaneous injection of SMMC7721 cells; (**B**) tumor growth graph; (**C**,**D**) statistical analyses of the dissected tumors for volume and weight. Black dots represent the LV–NC group, and red squares represent the 2041–OE group. (**E**) Nude mice were dissected and photographed 21 days after subcutaneous injection of HCCLM3 cells; (**F**) tumor growth graphs; (**G**,**H**) statistical analyses of the dissected tumors for volume and weight; (**I**) TUNEL assay to detect apoptosis rate in tumor tissues (100×); Black dots represent the sh–NC group, blue squares represent the 2041–sh1 group, and green triangles represent the 2041–sh2 group. (**J**) immunohistochemical staining to detect the Ki-67 content of tissues. * *p* < 0.05; ** *p* < 0.01; *** *p* < 0.001 indicated significant differences between groups.

The effect of LINC02041 on tumor growth in vivo was explored using a subcutaneous tumor formation experiment in nude mice. Nude mice were subcutaneously injected with SMMC7721 cells from the LINC02041 overexpression group and the control group, respectively, that had undergone stable transfection. Nude mice were euthanized after 21 days, and tumor tissues were dissected and statistically analyzed for volume and weight. Figure 3A displays representative tumor photographs. Tumors in nude mice injected with LINC02041-overexpressing cells were significantly larger than those in the control group. As shown in Figure 3B, the tumor growth rate in the nude mice injected with LINC02041-overexpressing cells was significantly higher than that in control mice. Quantitative measurements of tumor volume and weight further demonstrated that LINC02041 overexpression significantly promoted tumor growth (Figure 3C,D). HCCLM3 cells with stable LINC02041 knockdown and control cells were injected subcutaneously, and tumor tissues were dissected and statistically analyzed for their volume and weight. As shown in Figure 3E, tumors in nude mice injected with LINC02041 knockdown cells were significantly smaller than those in the control group, and tumor growth rate in the knockdown group was significantly slower than in the control group, as shown in (Figure 3F). There was also a significant difference in tumor volume and weight (Figure 3G,H). The results shown in Figure 3I indicate that the apoptosis rate in tumor tissues of mice in the knockdown group was significantly higher than in the control group. After immunohistochemical (IHC) staining of tumor tissues from the three groups of nude mice, we found that expression of Ki-67 protein was significantly reduced in tumors of nude mice injected with LINC02041 knockdown cells, as shown in Figure 3J. Collectively, these results demonstrate that LINC02041 promotes tumor growth in vivo in nude mice.

### 3.4. LINC02041 Promotes Glycolysis and Enhances Stem Cell-like Properties of HCC Cells

**Figure 4:**
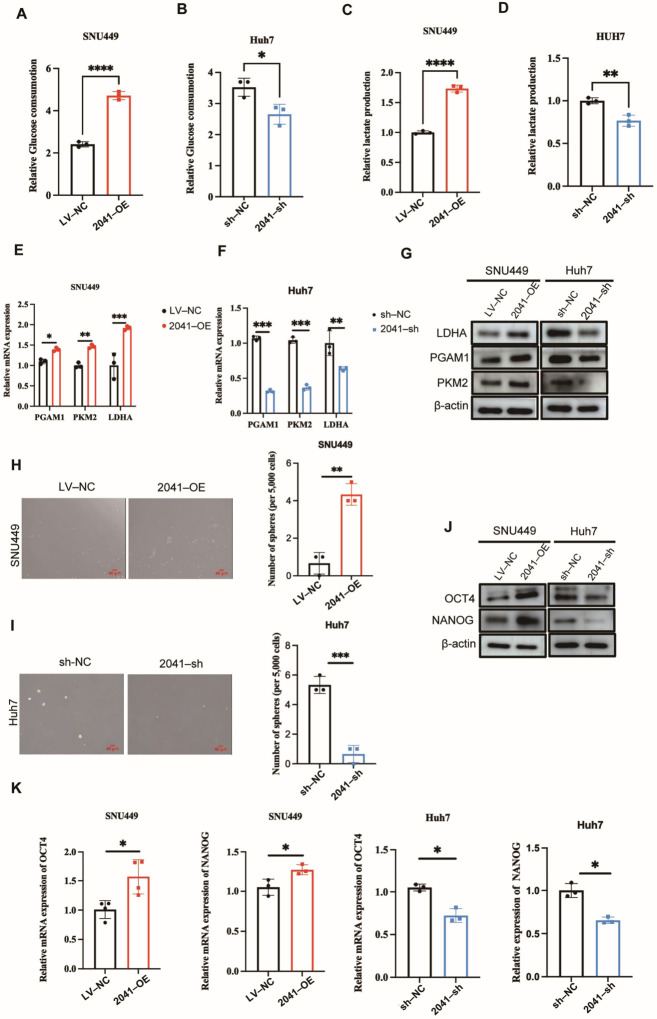
LINC02041 enhances the glycolysis and stem cell-like properties of HCC cells. (**A**–**D**) Detection of lactate production and glucose consumption in HCC cell lines overexpressing and knocking down LINC02041. (**E**,**F**) RT-qPCR results of metabolic enzymes of the glycolytic process in HCC cell lines overexpressing and knocking down LINC02041. (**G**) Western blot results of metabolic enzymes of the glycolytic process in HCC cell lines overexpressing and knocking down LINC02041. (**H**) Representative images of sphere formation in the LINC02041-overexpressing hepatocellular carcinoma cell line SNU449. (**I**) Representative images of sphere formation in the LINC02041-knockdown hepatocellular carcinoma cell line Huh7. (**J**) Western blot verification of stemness-related gene expression in cell lines with LINC02041 overexpression and knockdown. (**K**) RT-qPCR assay results for genes related to stemness in LINC02041-overexpressing and knockdown cell lines. In SNU449 cells, black dots represent the LV–NC group, and red squares represent the 2041–OE group. In Huh7 cells, black dots represent the sh–NC group, and blue squares represent the 2041–sh group. * *p* < 0.05; ** *p* < 0.01; *** *p* < 0.001, **** *p* < 0.0001.

To investigate the mechanism by which LINC02041 affects the progression of HCC, we performed transcriptome sequencing on cells with LINC02041 knockdown compared to the control group. GO functional enrichment analysis (Supplementary Figure S1A) showed that LINC02041 was significantly involved in regulating the metabolic processes of HCC cells. Furthermore, KEGG pathway analysis (Supplementary Figure S1B) indicated that LINC02041 may affect HCC cell energy metabolism by regulating the glycolytic pathway. By examining changes in glucose consumption and lactate production in SNU449 cells overexpressing LINC02041 and in Huh7 cells with LINC02041 knockdown, we found that LINC02041 increases glucose consumption and lactate production (Figure 4A–D). Given that glycolysis is regulated by various metabolic enzymes, we detected the expression of key glycolytic enzymes using RT-qPCR. As illustrated in Figure 4E,F, mRNA levels of PGAM1, PKM2, and LDHA were significantly upregulated in LINC02041-overexpressing SNU449 cells, whereas they were significantly downregulated in LINC02041 knockdown Huh7 cells. To further verify this phenomenon, we used Western blot analysis to detect the expression levels of LDHA, PGAM1, and PKM2 proteins in hepatocellular carcinoma cell lines with LINC02041 overexpression and knockdown. The results showed that the pattern of protein expression was consistent with that of the mRNA levels (Figure 4G). Together, these data demonstrate the positive regulatory effect of LINC02041 on the expression of glycolytic enzymes in HCC cell lines.

To investigate the effect of LINC02041 on the stemness of HCC cells, sphere-forming experiments were performed. The results showed that in SNU449 cells overexpressing LINC02041, the number and volume of hepatocellular carcinoma cell spheres were significantly higher than those of the control group (Figure 4H). Conversely, in Huh7 cells with LINC02041 knocked down, the number and volume of hepatocellular carcinoma cell spheres were significantly reduced compared with those of the control group (Figure 4I). Several tumor stemness markers in HCC, including OCT4 and NANOG, were then examined. The mRNAs and protein levels of OCT4 and NANOG were significantly increased in SNU449 cells overexpressing LINC02041 compared to the control group (Figure 4K,J), while in Huh7 cells with LINC02041 knocked down, the mRNA and protein levels of OCT4 and NANOG were significantly reduced compared to the control group (Figure 4K,J).

### 3.5. Inhibition of Stemness in LINC02041-Overexpressing Cells by the Glycolysis Inhibitor 2-Deoxy-D-Glucose (2-DG)

**Figure 5:**
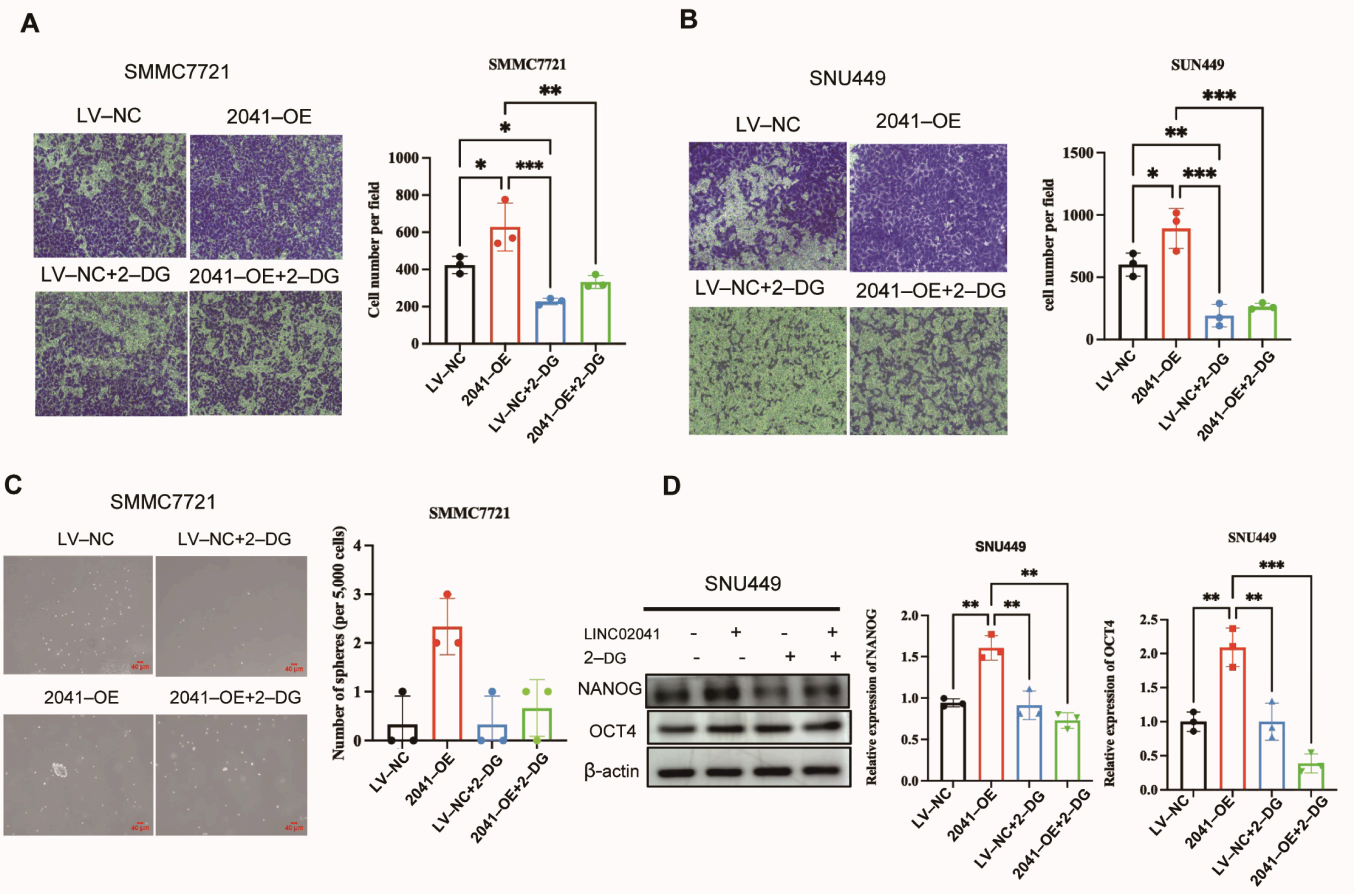
2-DG inhibits the migration and stemness of LINC02041-overexpressing cells. (**A**,**B**) Transwell assay was performed to detect the migratory capacity of HCC cells overexpressing LINC02041 compared to control cells and the effect of 2-DG treatment on cell migration. (**C**) Sphere formation assay to assess the number and size of spheres formed by HCC cell lines overexpressing LINC02041 versus control cell lines and the impact of 2-DG treatment. (**D**) RT-qPCR and Western blot analyses were conducted to verify the expression of stemness-related genes in LINC02041-overexpressing HCC cells treated with 2-DG. Black dots represent the LV–NC group, red squares represent the 2041–OE group, blue triangles represent the LV–NC + 2–DG group, and green inverted triangles represent the 2041–OE + 2–DG group. * *p* < 0.05, ** *p* < 0.01, *** *p* < 0.001.

We employed 2-deoxyglucose (2-DG) to suppress the Warburg effect. Migration assays were performed on LINC02041-overexpressing SMMC7721 and SNU449 cell lines following treatment with 2-DG. The results, as shown in Figure 5A,B, indicated that LINC02041 promotes HCC cell migration by enhancing glycolysis.

To investigate the effect of glycolysis on stemness of HCC cells overexpressing LINC02041, we performed a sphere-forming assay. The number of hepatocellular carcinoma cell spheres formed by SMMC7721 cells overexpressing LINC02041 was significantly higher than that of the control group. Additionally, the tumor spheres from the overexpression group were larger in volume compared to those from the control group. Upon treatment with 2-DG, both the number and volume of tumor spheres formed by LINC02041-overexpressing cells were markedly reduced, resulting in significantly lower sphere formation capacity compared to the untreated overexpression group (Figure 5C). We then used RT-PCR and Western blot to assess the levels of tumor stemness biomarkers in LINC02041-overexpressing cells with or without 2-DG treatment. In the untreated group, the expression levels of OCT4 and NANOG were significantly increased in LINC02041-overexpressing cells compared to the control group. However, following 2-DG treatment, the expression levels of OCT4 and NANOG in LINC02041-overexpressing cells showed no significant difference compared to the control group. Moreover, 2-DG-treated LINC02041-overexpressing cells showed significantly lower expression levels of OCT4 and NANOG than the untreated overexpression group (Figure 5D).

### 3.6. LINC02041 Enhances SRSF1 Protein Stability by Inhibiting Its Degradation

**Figure 6:**
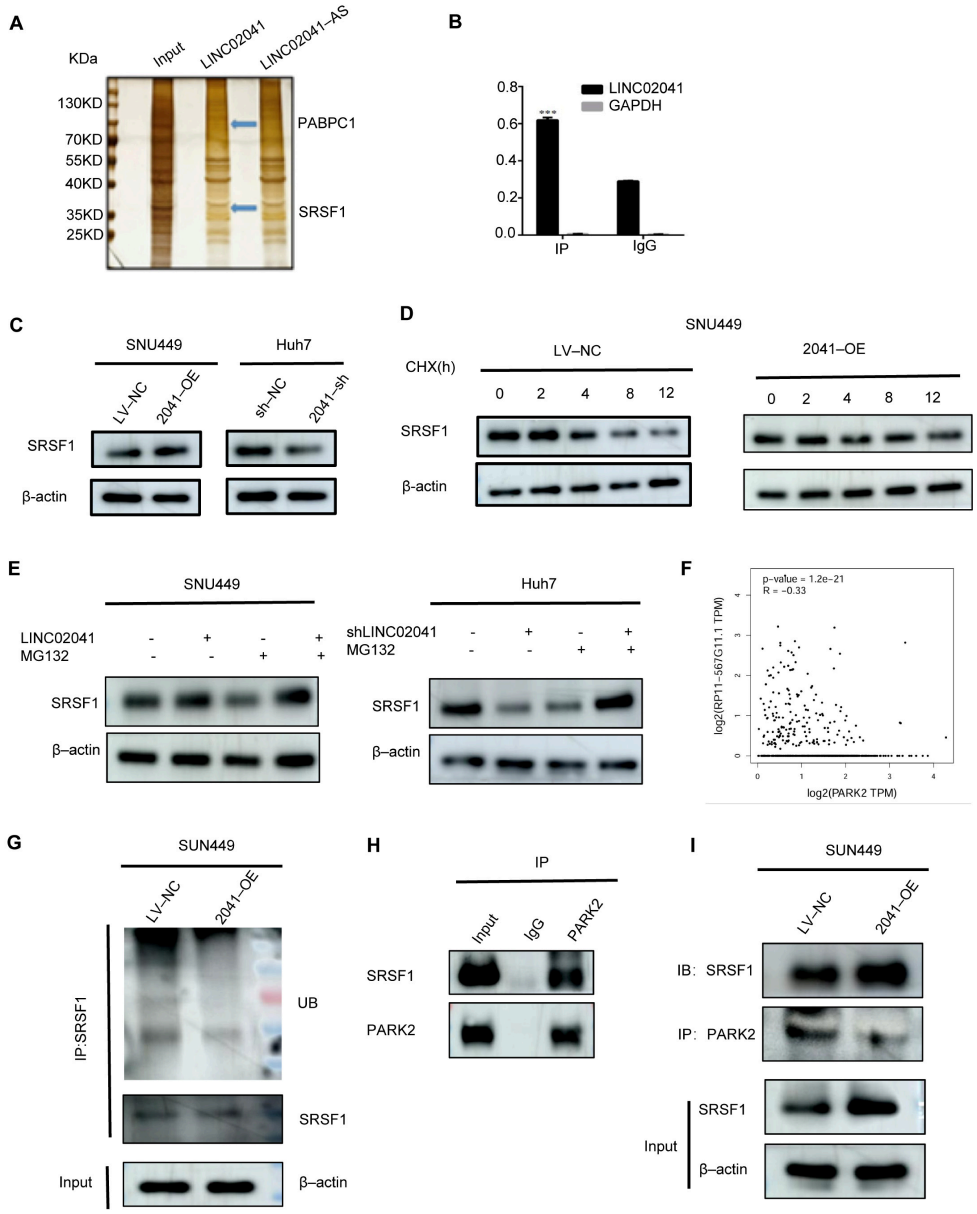
LINC02041 improves the stability of SRSF1 by preventing its degradation. (**A**) RNA pull-down detection showing protein silver staining results and mass spectrometry identification of proteins interacting with LINC02041. The arrows point to the positions of PABPC1 and SRSF1, respectively. (**B**) RNA immunoprecipitation (RIP) detection of SRSF1 interaction with LINC02041. (**C**) Western blot detection of SRSF1 expression following overexpression and knockdown of LINC02041 in hepatocellular carcinoma cell lines. (**D**) HCC cells overexpressing LINC02041 were treated with CHX, and SRSF1 protein degradation was detected by Western blot at 0, 2nd, 4th, and 6th hours. (**E**) SNU449 and Huh7 cells were treated with MG132, and SRSF1 protein degradation was detected by Western blot. (**F**) The correlation between LINC02041 and PARK2 was analyzed using the GEPIA database. (**G**) IP assay to detect the degree of ubiquitination of SRSF1 in SNU449 cells overexpressing LINC02041. (**H**) Co-IP verified the interaction between PARK2 and SRSF1. (**I**) Co-IP analysis of the effect of LINC02041 on the interaction between SRSF1 and PARK2. *** *p* < 0.001.

The PF00076 structural domain, strongly associated with LINC02041, was predicted using the catRAPID tool [31] available on the BioSignal website (Supplementary Figure S2A,B). Proteins containing the PF00076 domain were ranked according to their likelihood of interaction with LINC02041 (Supplementary Figure S2C). Subsequently, an RNA pull-down assay was performed in HCCLM3 cells using full-length LINC02041 labeled with luciferase, along with a control employing the reverse sequence of LINC02041. The proteins pulled down were then analyzed by mass spectrometry (Supplementary Figure S2D). Among the identified proteins were two predicted by catRAPID: poly (A)-binding protein cytoplasmic 1 (PABPC1) and serine/arginine-rich splicing factor 1 (SRSF1). Their positions in the silver-stained gel results are shown in Figure 6A. Western blot analysis using antibodies against PABPC1 and SRSF1 confirmed the interaction of these proteins with LINC02041 (Supplementary Figure S2E). Additionally, RNA immunoprecipitation (RIP) results demonstrated that SRSF1 interacted with LINC02041 (Figure 6B).

To explore the effect of LINC02041 on SRSF1, we detected SRSF1 protein levels by Western blot in SNU449 and Huh7 cells with LINC02041 overexpression or knockdown. These findings suggest that LINC02041 positively regulates SRSF1 expression (Figure 6C). We treated the SNU449 cell line with the protein synthesis inhibitor cycloheximide (CHX). As shown in Figure 6D, the SRSF1 protein levels decreased over time, but liver cancer cells overexpressing LINC02041 showed slower degradation of SRSF1 compared to the control group. The proteasome inhibitor MG132 was subsequently used to treat SNU449 cells overexpressing LINC02041 and Huh7 cells with LINC02041 knockdown, respectively. In the LINC02041 overexpression group, SRSF protein levels were significantly higher than those in the untreated control group without MG132 treatment. After MG132 treatment, the increase in SRSF1 protein levels was even more pronounced compared to the control group. In the LINC02041 knockdown group, the protein level of SRSF1 was decreased compared to the control group without MG132 treatment; however, after MG132 treatment, the decrease in SRSF1 protein levels caused by LINC02041 knockdown was reversed compared to the untreated group (Figure 6E). These results indicate that SRSF1 protein is mainly degraded through the ubiquitin–proteasome pathway. Subsequently, cells were treated with MG132, and the ubiquitination level of SRSF1 protein was detected by immunoprecipitation (IP). The results showed that the ubiquitination level of SRSF1 protein in LINC02041-overexpressing cells was significantly decreased compared to the control group (Figure 6G), indicating that LINC02041 inhibits the ubiquitination modification of SRSF1 protein.

Co-immunoprecipitation (Co-IP) assays were conducted in hepatocellular carcinoma cell lines using anti-PARK2 antibodies. Western blot analysis revealed an interaction between PARK2 and SRSF1 (Figure 6H). Data from the GEPIA database [32] reveal a strong negative correlation between LINC02041 (RP11-567G11.1) and PARK2 expression levels (Figure 6F). To further investigate this, we performed Co-IP experiments with antibodies against PARK2 in SNU449 cells overexpressing LINC02041 (Figure 6I). The results demonstrated that PARK2 protein levels were significantly reduced in the LINC02041-overexpressing group compared to controls, whereas SRSF1 protein levels were significantly elevated. These findings confirm an interaction between PARK2 and SRSF1 and show that LINC02041 overexpression is accompanied by reduced PARK2 abundance and increased SRSF1 abundance.

### 3.7. Overexpression of SRSF1 Rescues the Inhibitory Effects on the Proliferation, Migration, Glycolysis, and Stemness of HCC Cells Induced by LINC02041 Knockdown

**Figure 7:**
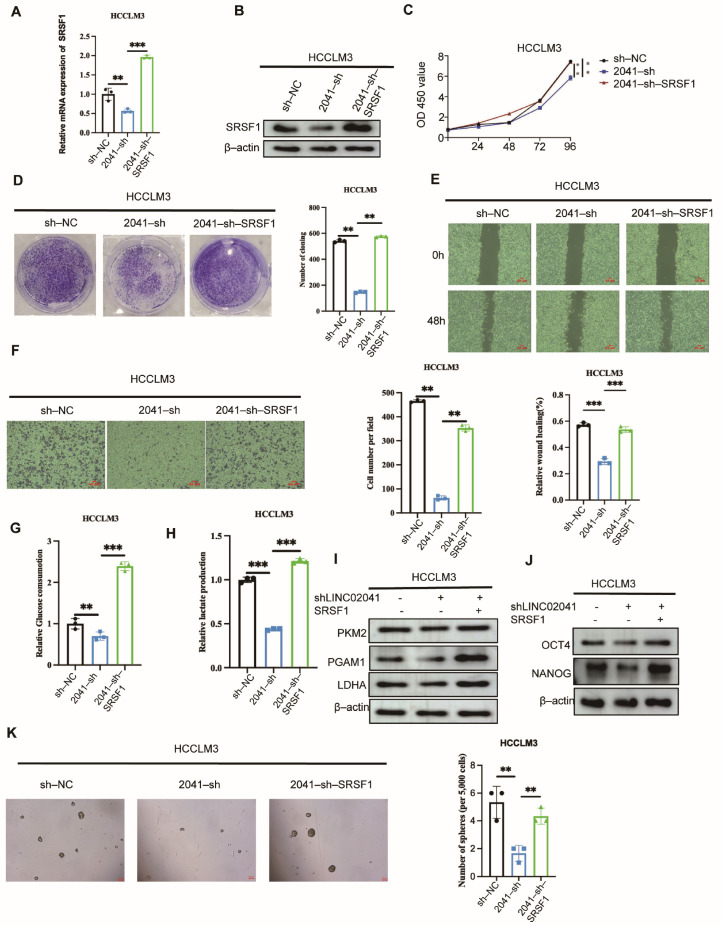
Overexpression of SRSF1 reverses the reduced proliferation, migration, glycolysis, and stemness suppressed by LINC02041 knockdown in HCC cells. (**A**) RT-qPCR analysis of SRSF1 mRNA levels in HCCLM3 cells with LINC02041 knockdown and SRSF1 overexpression. (**B**) Western blot analysis was used to detect SRSF1 protein levels in LINC02041 knockdown HCCLM3 cells and after overexpression of SRSF1. (**C**) CCK8 assay was conducted to assess the effect of SRSF1 overexpression on the proliferative ability of LINC02041 knockdown HCC cells in vitro. (**D**) Colony formation assay to evaluate the impact of SRSF1 overexpression on clonogenic capacity of LINC02041 knockdown HCC cells in vitro. (**E**) Wound healing assay to assess the effect of SRSF1 overexpression on the migration ability of LINC02041 knockdown HCC cells in vitro. (**F**) Transwell assay was conducted to evaluate the effect of SRSF1 overexpression on the migration ability of LINC02041 knockdown HCC cells in vitro. (**G**,**H**) Assessment of SRSF1 overexpression effects on lactate production and glucose consumption in LINC02041 low-expressing HCCLM3 cells. (**I**) Western blot assay was performed to detect the effects of SRSF1 overexpression on key glycolytic factors in LINC02041 low-expressing HCCLM3 cells. (**J**) Western blot assay was used to detect the effect of SRSF1 overexpression on key stemness-related factors in LINC02041 low-expressing HCCLM3 cells. (**K**) Sphere formation assay was conducted to evaluate the effect of SRSF1 overexpression on the number and size of spheres in LINC02041 low-expressing HCCLM3 cells. Black dots represent the sh–NC group, blue squares represent the 2041–sh group, and green triangles represent the 2041–sh–SRSF1 group. ** *p* < 0.01; *** *p* < 0.001.

Previous results indicated that LINC02041 promotes proliferation, glycolysis and stemness of HCC cells. To further investigate whether LINC02041 regulates these functions through SRSF1 in HCC, the expression of SRSF1 was restored in HCC cells subjected to LINC02041 knockdown. The rescue effect was assessed using RT-PCR and Western blot. As shown in Figure 7A,B), the mRNA and protein levels of SRSF1 in cells with LINC02041 knockdown and SRSF1 overexpression were significantly higher than those in cells with LINC02041 knockdown alone. CCK8 and clone formation assays were performed. Figure 7C shows that the OD450 values of cells in the LINC02041 knockdown group were significantly lower than those in the control group. In contrast, the OD450 values of LINC02041 knockdown cells overexpressing SRSF1 were significantly higher than those of cells with LINC02041 knockdown alone. Additionally, the colony formation assay results exhibited a similar trend (Figure 7D).

Wound healing and Transwell migration assays were used to investigate the effect of overexpressing SRSF1 on HCC cell migration in the context of LINC02041 knockdown. These assays aimed to verify whether SRSF1 overexpression could counteract the migration defects caused by LINC02041 knockdown. In the wound healing assay, the results, as shown in Figure 7E, indicated that LINC02041 knockdown significantly reduced the migration ability of HCCLM3 cells compared to control cells. When SRSF1 was overexpressed in LINC02041-knockdown cells, the migration ability was significantly enhanced compared to cells with LINC02041 knockdown alone. In the Transwell migration assay, as shown in Figure 7F, the number of HCCLM3 cells migrating through the membranes was significantly lower after LINC02041 knockdown than in control cells. However, LINC02041-knockdown cells overexpressing SRSF1 showed a significantly increased number of migrated cells compared to cells with LINC02041 knockdown alone. These results indicate that overexpression of SRSF1 can reverse the inhibitory effect of LINC02041 knockdown on HCC cell migration.

Knocking down LINC02041 inhibited glycolysis. Furthermore, we investigated whether SRSF1 affects glycolysis. In HCC cells with LINC02041 knockdown, both glucose consumption and lactate production were significantly reduced compared with the control group. However, these levels were significantly higher in cells overexpressing SRSF1 in the context of LINC02041 knockdown (Figure 7G,H). Additionally, Western blotting was performed to verify the expression of glycolytic metabolism-related proteins such as PKM2, PGAM1, and LDHA. The expression of these proteins in the LINC02041 knockdown group was significantly lower than that in the control group, but their expression in the LINC02041 knockdown plus SRSF1 overexpression group was markedly restored compared to the LINC02041 knockdown group (Figure 7I). Western blot also confirmed the expression patterns of stemness-related proteins OCT4 and NANOG (Figure 7J). In the sphere-forming assay, HCC cells with LINC02041 knockdown formed significantly fewer and smaller spheres than the control group. However, overexpression of SRSF1 in these knockdown cells increased both the number and size of spheres compared to LINC02041 knockdown alone (Figure 7K).

### 3.8. STAT3 Transcriptionally Regulates LINC02041 Expression, and Overexpression of SRSF1 Reverses the Tumor Growth Inhibition Induced by LINC02041 Knockdown in Mice

**Figure 8:**
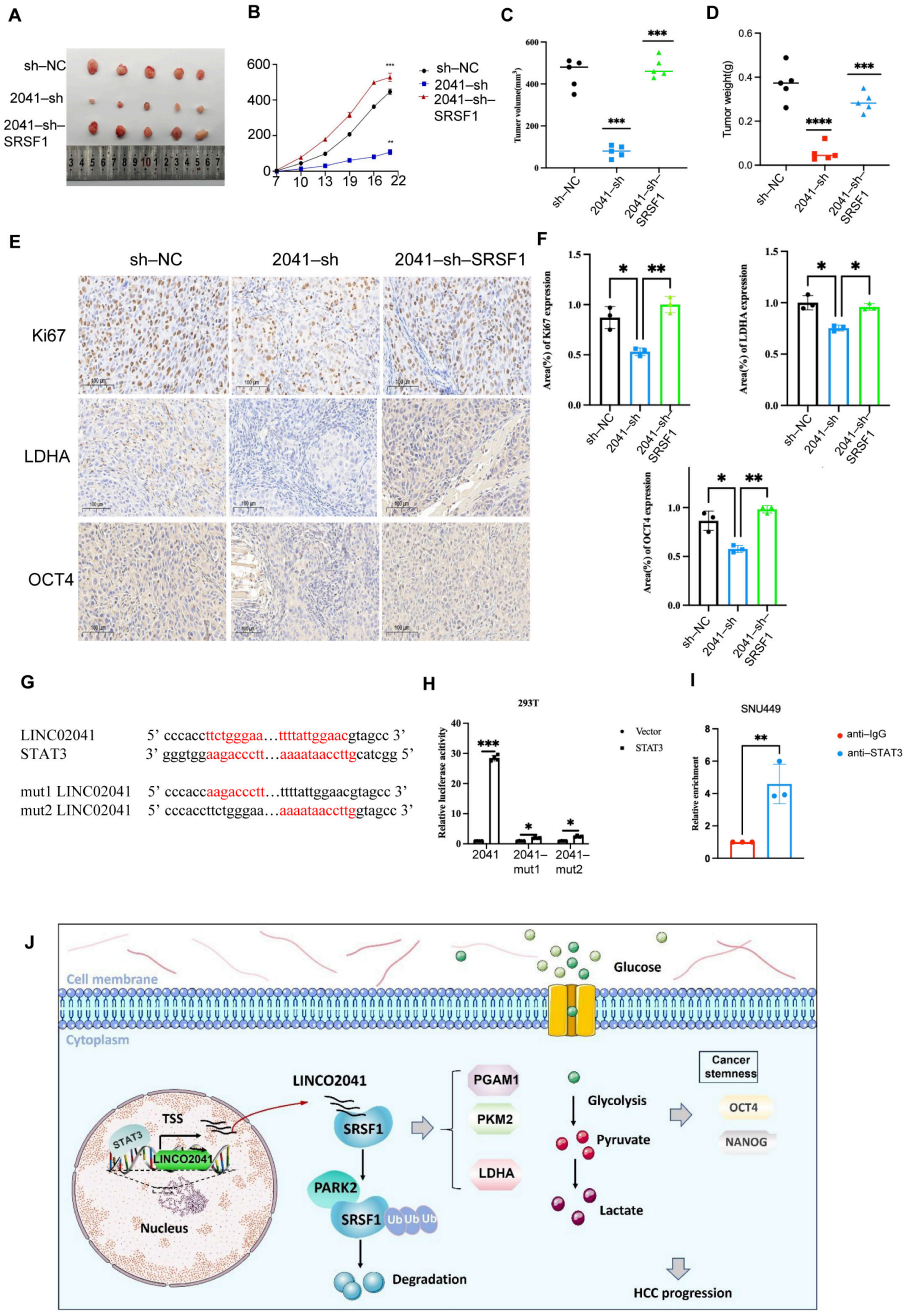
Overexpression of SRSF1 reverses tumor growth inhibition in mice caused by LINC02041 knockdown. STAT3 transcriptionally regulates the expression of LINC02041. (**A**) Tumors were removed from nude mice by dissection 21 days after subcutaneous injection of HCCLM3 cells. (**B**) Tumor growth graphs following tumor formation in nude mice. (**C**) Statistical analysis of the volume of tumors dissected from nude mice after 21 days. (**D**) Statistical analysis of the weight of tumors dissected from nude mice after 21 days. (**E**) Immunohistochemical staining of tissues to detect the expression levels of Ki-67, LDHA, and OCT4. Black dots represent the sh–NC group, blue squares represent the 2041–sh group, and green trian-gles represent the 2041–sh–SRSF1 group. (**F**) Analysis of the correlation between LINC02041 and STAT3 using the GEPIA database. (**G**) Design of mutation sites for the binding of STAT3 to the LINC02041 promoter. (**H**) Dual-luciferase reporter gene assay to validate the binding interaction between the LINC02041 promoter and STAT3. (**I**) ChIP assay to verify the binding interaction between the LINC02041 promoter and STAT3. (**J**) A diagram illustrating the mechanism by which LINC02041 promotes stemness through glycolysis. * *p* < 0.05; ** *p* < 0.01; *** *p* < 0.001; **** *p* < 0.0001.

To further explore its role in vivo, we performed subcutaneous tumor formation experiments in nude mice. The results showed that the tumor volume and weight of the nude mice in the LINC02041 knockdown group were significantly smaller than those in the control group. In contrast, the tumor volume and weight in the LINC02041 knockdown group with SRSF1 overexpression were significantly increased compared to the LINC02041 knockdown group (Figure 8A). Tumor volume was measured every three days starting from day 7 after HCC cell injection, and growth curves were plotted (Figure 8B). The data showed that tumors in the LINC02041 knockdown group grew significantly more slowly than those in the control group, while tumors in the LINC02041 knockdown group with SRSF1 overexpression grew faster than those in the LINC02041 knockdown group. These findings were further confirmed by tumor volume and weight measurements (Figure 8C,D). Immunohistochemical (IHC) staining showed that the expression of Ki-67, LDHA and OCT4 proteins in tumor tissues from the LINC02041 knockdown group was significantly reduced compared with the control group. However, overexpression of SRSF1 in the LINC02041 knockdown group reversed the reduction in the expression of these proteins (Figure 8E). These results suggest that SRSF1 overexpression can reverse the inhibition of tumor growth caused by LINC02041 knockdown in mice.

A strong correlation between LINC02041 (RP11-567G11.1) and the expression of the transcription factor STAT3 was identified via the GEPIA database [32] (Supplementary Figure S2F).

The most probable STAT3 binding site within the LINC02041 promoter was predicted using the JASPAR online database [33]. Two mutant LINC02041 vectors containing specific mutations were constructed for the dual-luciferase reporter gene experiment. These mutation sites are shown in Figure 8G. STAT3 binding to the LINC02041 promoter was confirmed by the dual luciferase reporter gene experiment (Figure 8H). Furthermore, ChIP experiments demonstrated that STAT3 binds to the LINC02041 promoter region (Figure 8I). The proposed mechanistic model summarizing these findings is illustrated in Figure 8J.

## 4. Discussion

This study extends the known functions of LINC02041 by associating it with glycolytic activity and stem-like properties in HCC. Our findings support a regulatory model in which STAT3 activates LINC02041 transcription, subsequently leading to LINC02041 interaction with SRSF1 and enhanced protein stability by reducing ubiquitin-mediated degradation. SRSF1 restoration also partially reversed the effects of LINC02041 knockdown on phenotypes associated with malignancy, glycolysis, and stemness. Together, these findings connect LINC02041-associated regulation of SRSF1 stability with metabolic and stemness-related phenotypes, although the precise involvement of PARK2 requires further functional validation.

Previous studies demonstrated that LINC02041, also annotated as RP11-567G11.1, promotes migration and invasion in pancreatic cancer and renal cell carcinoma [24,25]. The present study extends these observations to HCC and further links LINC02041 to SRSF1 protein stability, glycolytic activity, and stem-like properties. Thus, the principal advance of this work is not merely the identification of an additional cancer-associated lncRNA, but the mechanistic link between lncRNA-dependent protein stabilization and metabolic regulation in HCC.

Metabolic adaptation, particularly enhanced glycolysis, supports tumor growth, cellular plasticity, and stem-like properties [9,11,12,13]. Although several oncogenic pathways regulate glycolytic metabolism [34,35,36], how lncRNAs coordinate glycolytic activity with stemness in HCC remains incompletely understood. Our findings position LINC02041 within this regulatory framework by demonstrating an association between its expression and glycolytic activity as well as stem-like phenotypes.

In this study, LINC02041 overexpression increased glucose consumption, lactate production, and the expression of glycolysis enzymes, including PKM2, PGAM1, and LDHA. Treatment with 2-deoxy-D-glucose (2-DG) partially attenuated LINC02041-induced sphere formation and stemness-marker expression. These findings suggest that glycolytic reprogramming functionally contributes to LINC02041-mediated stem-like properties. However, because 2-DG is not specific to glycolysis [37] and only partially reversed the observed phenotypes, complementary genetic and metabolic interventions are required to confirm this functional relationship.

Cancer stem cells (CSCs) represent a subpopulation of tumor cells characterized by self-renewal capacity, differentiation potential, and tumor-initiating ability, which contribute to tumor recurrence, metastasis, and therapeutic resistance [5,6]. Increasing evidence indicates that metabolic adaptation, particularly enhanced glycolysis, is closely associated with the maintenance of CSC properties [14,38]. In addition to lncRNAs, other non-coding RNAs, particularly microRNAs, have been implicated in CSC regulation through modulation of stemness-associated genes and signaling pathways. For example, miR-128 and miR-600 have been reported to regulate CSC-like properties through the BMI-1 and WNT signaling pathways, respectively [39,40]. Consistent with these findings, our study demonstrated that LINC02041 promotes stemness characteristics in HCC cells, while glycolytic inhibition partially reverses these effects. These results suggest that LINC02041 regulates HCC stemness through metabolic reprogramming, thereby providing a potential mechanistic link between lncRNA regulation and cancer metabolism.

SRSF1 is a representative member of the SR protein family and was among the first splicing factors identified as a proto-oncogene [26]. Previous studies have demonstrated that SRSF1 promotes tumorigenesis through the regulation of alternative splicing, proliferation, apoptosis, and metastatic signaling pathways [26,27]. In HCC, SRSF1 has been reported to regulate SRA1 alternative splicing and promote tumor invasion [27]. In this study, we demonstrate that LINC02041 interacts with SRSF1, and LINC02041 overexpression led to reduced SRSF1 ubiquitination and increased SRSF1 protein stability.

Furthermore, our results suggest that PARK2 may modulate SRSF1 stability. PARK2, a RING-between-RING (RBR)-type E3 ubiquitin ligase, regulates substrate ubiquitination and has also been implicated in metabolic regulation, including the modulation of glycolytic activity [41]. In our study, PARK2 interacts with SRSF1, and LINC02041 negatively regulates PARK2 expression. These findings suggest that LINC02041 may enhance SRSF1 stability, at least partially, through modulation of PARK2-mediated ubiquitination. This provides a potential mechanism by which lncRNAs regulate oncogenic RNA-binding proteins via post-translational mechanisms.

STAT3 is a critical transcription factor involved in tumor initiation and progression that is frequently activated in human malignancies, including HCC [42,43]. Persistent STAT3 activation promotes tumor development through the regulation of genes involved in proliferation, survival, metabolism, and stemness maintenance [42]. Previous studies have demonstrated that STAT3 regulates oncogenic lncRNAs in HCC, suggesting that non-coding RNA networks represent important downstream effectors of STAT3 signaling [43]. In the present study, we demonstrated that STAT3 directly binds to the promoter region of LINC02041 and transcriptionally activates its expression. These findings identify LINC02041 as a novel downstream effector of STAT3 signaling and expand the current understanding of STAT3-mediated regulatory networks in HCC.

From a therapeutic perspective, our findings may provide a rationale for targeting the LINC02041-associated regulatory network in HCC. Given that LINC02041 promotes glycolytic reprogramming and CSC-like properties, inhibition of LINC02041 may enhance the efficacy of currently approved therapies, including sorafenib and lenvatinib, by reducing metabolic adaptability and stemness-associated resistance. Metabolic reprogramming and cancer stem cell properties have been increasingly recognized as important contributors to therapeutic resistance in HCC [6,15]. However, this hypothesis requires further validation through drug-resistant HCC models and combination treatment experiments.

In addition, our identification of STAT3 as an upstream transcriptional regulator of LINC02041 suggests that pharmacological inhibition of STAT3 signaling offers a potential strategy to suppress the STAT3/LINC02041-associated oncogenic program. STAT3 inhibitors have demonstrated antitumor activity in preclinical cancer models and may offer therapeutic potential for tumors characterized by persistent STAT3 activation and aggressive biological behavior [42,43]. Furthermore, because LINC02041 enhances SRSF1 protein stability by reducing ubiquitin-mediated degradation, modulation of the ubiquitin–proteasome system may represent another potential avenue for therapeutic exploration. Nevertheless, the clinical relevance of proteasome inhibitors in HCC remains to be established, and further studies are required to determine whether targeting the STAT3/LINC02041/SRSF1 axis can improve therapeutic responses in vivo.

From a clinical perspective, our findings suggest that LINC02041 represents a potential molecular biomarker and therapeutic target for HCC. However, several limitations should be acknowledged. First, the clinical validation cohort was relatively small, comprising just 12 paired HCC and adjacent non-tumor tissue samples. Validation in larger independent cohorts is warranted to establish the clinical significance and prognostic value of LINC02041. Second, additional clinically relevant models are required to further evaluate the therapeutic potential of targeting LINC02041. Finally, although our study identified the role of LINC02041 in modulating SRSF1 stability, the precise molecular mechanisms underlying LINC02041-mediated regulation of PARK2/SRSF1 ubiquitination warrant further investigation.

## 5. Summary

LINC02041 was upregulated in the TCGA-LIHC cohort and in the examined clinical tissue cohort. In vitro assays and xenograft models showed that LINC02041 promoted HCC cell proliferation, migration, invasion, and tumor growth. LINC02041 also enhanced aerobic glycolysis and stem-like properties in HCC cells. The partial attenuation of these effects by glycolytic inhibition suggested that glycolytic reprogramming contributes to LINC02041-mediated stem-like phenotypes. Mechanistically, LINC02041 interacted with SRSF1 and increased its protein stability by reducing ubiquitin-mediated degradation. SRSF1 overexpression partially rescued the impaired proliferation, migration, glycolytic activity, and stem-like properties caused by LINC02041 knockdown. Furthermore, STAT3 bound to the LINC02041 promoter and transcriptionally activated its expression. Collectively, these findings support a STAT3/LINC02041/SRSF1 regulatory axis that contributes to HCC progression and identify LINC02041 as a candidate biomarker and therapeutic target requiring further clinical validation.

## Data Availability

The original contributions presented in this study are included in the article/Supplementary Material. Further inquiries can be directed to the corresponding authors.

## Supplementary Materials

The following supporting information can be downloaded at: https://www.mdpi.com/article/10.3390/cells15151350/s1, Figure S1: Enrichment analysis of the differentially expressed genes identified by transcriptome of LINC02041 knockdown versus the control cell, including GO (A) and KEGG pathway analysis (B). Figure S2: (A) The catRAPID website predicts the number of structural domains of each protein interacting with LINC02041. (B) The catRAPID website predicts structural domains that most strongly interact with LINC02041. (C) The catRAPID website predicts the genes most likely to interact with LINC02041. (D) Mass spectrometry analysis of proteins pulled down by RNA after LINC02041 RNA pull down. (E) The correlation between STAT3 and LINC02041 was analyzed using the GEPIA database. Table S1: Primer sequences used for RT-qPCR and ChIP-qPCR analyses. Table S2: List of abbreviations.
