# Supplementary material for: The LINC02041/SRSF1 Axis Facilitates Aerobic Glycolysis and Stemness Maintenance in Hepatocellular Carcinoma
Source: Cells. 2026 Jul 27;15(15):1350. doi: 10.3390/cells15151350 (PMC13465720; doi:10.3390/cells15151350)
Supplement: Supplementary file 1 [file cells-15-01350-s001.zip › Supplementary Files/Supplementary figures.pdf]

Supplementary figure S1

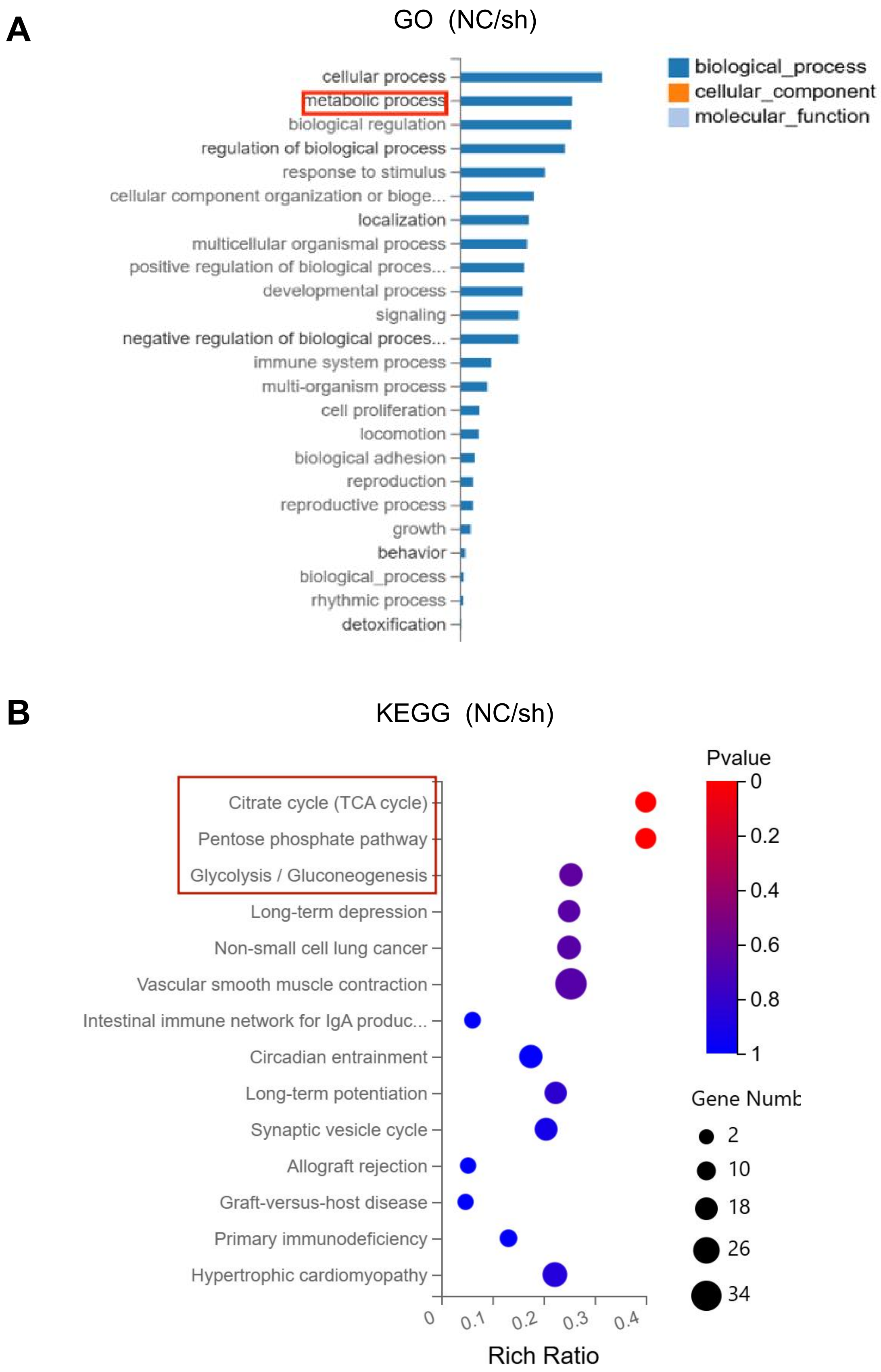

Supplementary figure S1:
Enrichment analysis of the differentially expressed genes identified by transcriptome of LINC02041 knockdown versus the control cell, including GO (A) and KEGG pathway analysis (B).

Supplementary figure S2

A

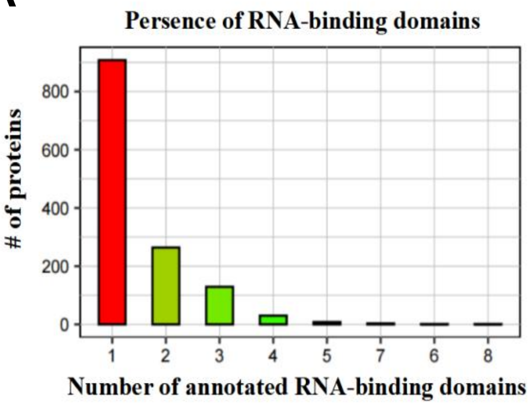

B

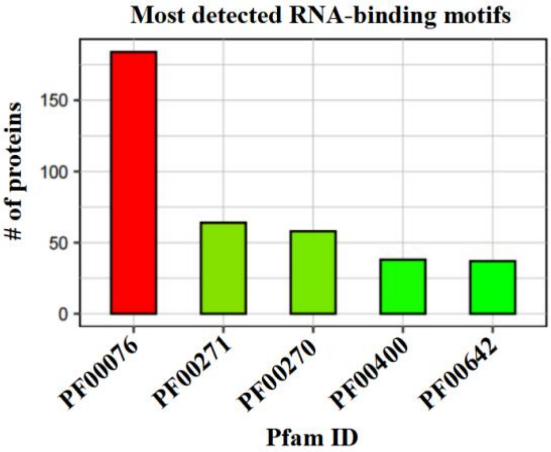

C

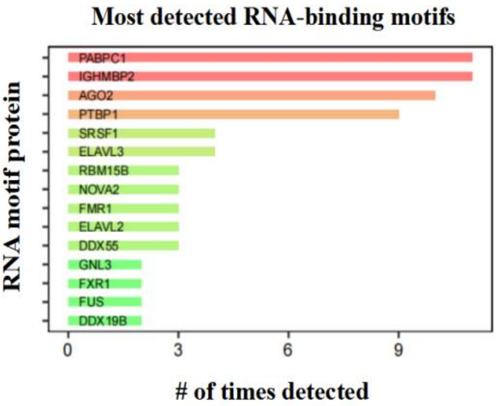

D

蛋白编号	基因名称	蛋白打分值	序列覆盖率（%）	特异性肽段数目	肽段数目	二级谱图数目
Q07955	SRSF1	156	28	6	7	7
P11940	PABP C1	119	4	2	2	2

E

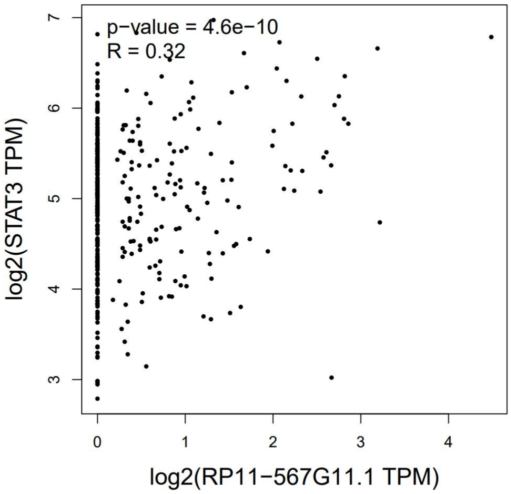

Supplementary figure S2:
(A) The catRAPID website predicts the number of structural domains of each protein interacting with LINC02041. (B) The catRAPID website predicts structural domains that most strongly interact with LINC02041. (C) The catRAPID website predicts the genes most likely to interact with LINC02041. (D) Mass spectrometry analysis of proteins pulled down by RNA after LINC02041 RNA pull down. (E) The correlation between STAT3 and LINC02041 was analyzed using the GEPIA database.
