# Supplementary material for: The LINC02041/SRSF1 Axis Facilitates Aerobic Glycolysis and Stemness Maintenance in Hepatocellular Carcinoma
Source: Cells. 2026 Jul 27;15(15):1350. doi: 10.3390/cells15151350 (PMC13465720; doi:10.3390/cells15151350)
Supplement: Supplementary file 1 [file cells-15-01350-s001.zip › Supplementary Files/Supplementary Tables.pdf]

**Table S1.** Primer sequences used for RT-qPCR and ChIP-qPCR analyses.

**RT-qPCR**

| Gene | Forward primer（5’-3’） | Reverse primer（5’-3’） |
|---|---|---|
| SRSF1 | CCGCAGGGAACAACGATTG | GCCGTATTTGTAGAACACGTCCT |
| β-actin | CGGTATCGTTCTGGACTCCG | GCGGTGGTGGTGAAAGAGTA |
| LINC2041 | TCAAGAATGGACGGCTTCAAGGTG | GCAGGTCTGAGTATGAGGTGAGTG |
| PKM2 | CCTGATAGCTCGTGAGGCTG | TTGAGGCTCGCACAAGTTCT |
| LDHA | TTGTCTCTGGCAAAGTGGAT | ACCGCTTCCAATAACACGGT |
| PGAM1 | GAAGCAAAACGCAGGACAGT | GGATCGCTCTCTTCTGCACT |
| NANOG | TTTGTGGGCCTGAAGAAAACT | AGGGCTGTCCTGAATAAGCAG |
| OCT4 | CTGGGTTGATCCTCGGACCT | CCATCGGAGTTGCTCTCCA |

**ChIP-qPCR**

| Target region | Forward primer（5’-3’） | Reverse primer（5’-3’） |
|---|---|---|
| LINC02041 promoter | ACTCCCTTCTCTATTGTGTT | TTTTCTTCTTTTGATCTTTT |
| SOCS3 promoter | CGCGCACAGCCTTTCAGT | CCCCCGATTCCTGGAACT |
| LINC02041 distal region | GGTGGATGCTATTGCAGCTC | TGAATCTGGGCAGCTACAGG |

**Table S2.** List of abbreviations

| English abbreviation | English full name |
|---|---|
| HCC | Hepatocellular carcinoma |
| cDNA | Complementary DNA |
| DMSO | Dimethyl sulfoxide |
| BCA | Bicinchoninic acid |
| OCT4 | Octamer-binding transcription factor 4 |
| NANOG | Nanog homeobox |
| IHC | Immunohistochemistry |
| APS | Ammonium persulfate |
| CCK-8 | Cell Counting Kit-8 |
| EMT | Epithelial-mesenchymal transition |
| LDHA | Lactate dehydrogenase A |
| PKM2 | Pyruvate kinase M2 |
| PGAM1 | Phosphoglycerate Mutase 1 |
| DEPC | Diethyl pyrocarbonate |
| BSA | Bovine serum albumin |
| ECL | Electrochemiluminescence |

| | |
|---|---|
| Tris | Tris(hydroxymethyl)aminomethane |
| FBS | Fetal bovine serum |
| SRSF1 | serine/arginine-rich splicing factor 1 |
| LINC RNA | Long intergenic non-coding RNA |
| RT-qPCR | real-time Quantitative PCR |
| STAT3 | Signal transducer and activator of transcription 3 |
| PMSF | Phenylmethylsulfonyl fluoride |
| PARK2 | Parkin RBR E3 Ubiquitin Protein Ligase |
| PVDF | Polyvinylidene fluoride |
| PBS | Phosphate-buffered saline |
| SDS | Sodium dodecyl sulfate |
| MOI | Multiplicity of infection |
| TEMED | N,N,N',N'-Tetramethylethylenediamine |
| lncRNA | Long non-coding RNA |
| β-actin | Beta-actin |
